# Correlation between immune-related adverse events and therapeutic effects of nivolumab in patients with malignant pleural mesothelioma

**DOI:** 10.1186/s12890-021-01746-6

**Published:** 2021-11-15

**Authors:** Hiroto Yoneda, Hiroshi Nokihara, Atsushi Mitsuhashi, Ryohiko Ozaki, Yohei Yabuki, Hirokazu Ogino, Kenji Otsuka, Yasuhiko Nishioka

**Affiliations:** Department of Respiratory Medicine and Rheumatology, Graduate School of Biomedical Sciences, Tokushima University, 3-18-15, Kuramoto-cho, Tokushima, 770-8503 Japan

**Keywords:** Immune-related adverse events, Malignant pleural mesothelioma, Nivolumab, Therapeutic effect

## Abstract

**Background:**

Nivolumab is used for the treatment of malignant pleural mesothelioma (MPM). However, immune-related adverse events (irAEs) occur in patients treated with nivolumab. Several studies have reported the correlation between irAEs and therapeutic effects of immune checkpoint inhibitor, but none have reported the correlation in MPM. Here we report a retrospective study which shows the correlation between irAEs and therapeutic effects of nivolumab in patients with MPM.

**Methods:**

This study included patients treated with nivolumab at Tokushima University Hospital from February 2009 to September 2021. We retrospectively reviewed the medical records to evaluate the several clinical factors, such as the presence or absence of irAEs, their severities, progression-free survival (PFS), overall survival (OS) or objective response to the treatment.

**Results:**

Eleven patients received treatment with nivolumab. Objective response rate was 18.2% and the disease control rate was 90.9%. Median PFS was 6.8 months (95% confidence interval, 1.3 to 11.9 months) and median OS was 15.2 months (95% confidence interval, 8.9 to 21.5 months). IrAEs occurred in eight patients (72.7%), and grade ≥ 2 irAEs occurred in six patients (54.5%). PFS and OS were significantly longer in the grade ≥ 2 irAEs group than in grade < 2 irAEs group (median PFS 13.6 vs. 3.8 months, *p* = 0.0093; median OS not reached vs. 8.6 months, *p* = 0.0108).

**Conclusions:**

This is the first study to report the correlation between irAEs and therapeutic effects in patients with MPM. Because the presence of irAEs may be associated with a favorable clinical outcome, early detection and appropriate management of irAEs will increase the therapeutic benefits to patients.

## Background

Malignant pleural mesothelioma (MPM) is a rare tumor, but it is an aggressive tumor and has a poor prognosis. The median overall survival (OS) is reported to be approximately seven months without any treatments [1], and it is also reported to be 6 to 18 months even with the appropriate treatments, regardless of the therapeutic modalities [2–4]. Many patients with MPM are not offered surgery due to advanced stage, old age, comorbidities, or poor performance status, and are instead considered to be palliative chemotherapy. The combination of cisplatin and pemetrexed is a standard first-line treatment for unresectable MPM [5]. Nivolumab, an anti-programmed cell death protein 1 (PD-1) antibody, has showed an encouraging clinical benefits as a second- or third- line treatment [6, 7], and has been approved in Japan since 2018.

The treatment with nivolumab may lead to immune-related adverse events (irAEs), which sometimes results in the interruption or discontinuation of the treatment [8]. Previous reports in melanoma, non-small-cell lung cancer, and gastric cancer patients have shown that the presence of irAEs with nivolumab were positively associated with their progression-free survival (PFS) and OS [9–13]. However, the correlation between irAEs and outcome of nivolumab for patients with MPM is still unknown. As such, we conducted the retrospective study to investigate whether irAEs are associated with clinical efficacies of nivolumab in MPM.

## Methods

### Participants

**Fig. 1:**
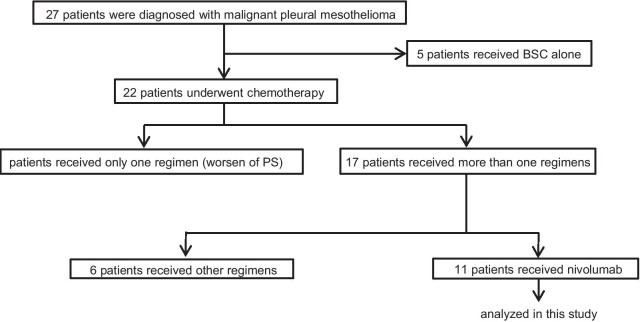
Flow diagram for study participants. *BSC* best supportive care, *PS* performance status, *PD* progressive disease

Patients, who were diagnosed as MPM in the Department of Respiratory Medicine and Rheumatology at Tokushima University Hospital from February 1st, 2009 to September 30th, 2021, were retrospectively analyzed. Twenty-seven patients were diagnosed with MPM (Fig. 1). Five patients received best supportive care alone, and 22 patients received chemotherapy. 17 of 22 patients received two or more regimens, and eleven patients were treated with nivolumab, and we analyzed these eleven patients in this study. This study was performed in accordance with the Declaration of Helsinki and was approved by the Institutional review board.

### Data collection

We examined several clinical factors including age, gender, Eastern Cooperative Oncology Group (ECOG) performance status (PS), histology, clinical stage, presence or absence of irAEs, the severities of irAEs, PFS, OS and objective response to the treatment. The data were collected retrospectively from the medical records in our hospital.

### Treatment and assessment

Nivolumab was administered intravenously at a dose of 240 mg/body every two weeks. Nivolumab was administered until disease progression or unacceptable adverse events. Adverse events were assessed according to the National Cancer Institute-Common Toxicity Criteria for Adverse Events (NCI-CTCAE) version 5.0. Clinical responses to the treatment were categorized as either complete response (CR), partial response (PR), stable disease (SD), or progressive disease (PD) according to the modified Response Evaluation Criteria in Solid Tumors (mRECIST) or the Response Evaluation Criteria in Solid Tumors (RECIST) version 1.1. The target lesions in pleura were measured uni-dimensionally as the largest tumor thickness perpendicular to the chest wall or mediastinum according to mRECIST [14]; those in nonpleura were measured according to RECIST version 1.1. CR was defined as the disappearance of all target lesions with no evidence of tumor elsewhere, and PR was defined as at least a 30% reduction in the total tumor measurement. A confirmed response required a repeat observation on two occasions 4 weeks apart. PD was defined as an increase of at least 20% in the total tumor measurement over the nadir measurement, or the appearance of one or more new lesions. Patients with SD were those who fulfilled the criteria for neither PR nor PD. Objective response rate (ORR) was defined as the proportion of CR and PR, and disease control rate (DCR) was defined as that of CR, PR and SD. PFS was defined as the period from the start of treatment with nivolumab to the date of disease progression. OS was defined as the period from the start of treatment with nivolumab to death or loss of follow-up.

### Statistical analysis

PFS and OS were estimated by the Kaplan-Meier method, and their statistical differences were analyzed by the Log-rank test. The statistical analyses were performed using GraphPad Prism version 7 (GraphPad Software, La Jolla, California USA). In this analysis, a *p* value of < 0.05 was considered to indicate a significant difference.

## Results

### Patient characteristics

Patient characteristics are shown in Table 1. Among the total eleven patients, eight patients (72.7%) were male and three patients (27.3%) were female, and median age was 72.0 (56–84) years. Six of eleven patients (54.5%) have asbestos inhalation history. All patients were histologically epithelial type. All patients received a combination chemotherapy of platinum (five received cisplatin and six received carboplatin) and pemetrexed as a first-line treatment. Ten patients received nivolumab as a second-line and one received as a third-line treatment.

**Table 1:** Patient characteristics

No. of patients	11	
Gender (%)		
Male	8	(72.7)
Female	3	(27.3)
Age (years)		
Median (range)	72	(56–84)
ECOG performance status (%)		
0	4	(36.4)
1	6	(54.5)
2	1	(9.1)
Smoking status (%)		
Never	4	(36.4)
Ex/current	7	(63.6)
Asbestos inhalation history (%)		
+	6	(54.5)
−	5	(45.5)
Histology (%)		
Epithelial type	11	(100.0)
1st line regimen		
CDDP + PEM	5	(45.5)
CBDCA + PEM	6	(54.5)
Regimen line (%)		
2nd	10	(90.9)
3rd	1	(9.1)

*CDDP* cisplatin, *PEM* pemetrexed, *CBDCA* carboplatin

### Effects of nivolumab in previously treated MPM patients

**Fig. 2:**
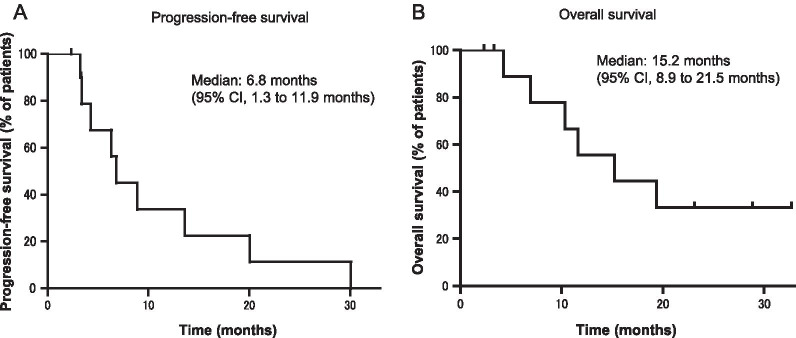
Kaplan–Meier survival curve of progression free survival (**A**) and overall survival (**B**) in previously treated MPM patients

Regarding the best overall response, PR was 18.2%, SD was 72.7%, and PD was 9.1%. The ORR was 18.2% and the DCR was 90.9% (Table 2). Median follow-up time was 15.2 months. Median PFS was 6.8 months (95% confidence interval [CI], 1.3 to 11.9 months) and median OS was 15.2 months (95% CI, 8.9 to 21.5 months) (Fig. 2).

**Table 2:** Anti-tumor effect of nivolumab in previously treated malignant pleural mesothelioma patients

No. of patients	11	
Best overall response (%)		
CR	0	(0.0)
PR	2	(18.2)
SD	8	(72.7)
PD	1	(9.1)
ORR	18.2%	
DCR	90.9%	

*CR* complete response, *PR* partial response, *SD* stable disease, *PD* progressive disease, *ORR* objective response rate, *DCR* disease control rate

### Onset of irAEs

The irAEs were observed in total eight patients (72.7%), and grade ≥ 2 irAEs were found in six patients (54.5%) (Tables 3, 4). Two patients were diagnosed with pneumonitis of irAEs by their CT imaging patterns; organizing pneumonia pattern in case 1 and nonspecific interstitial pneumonia pattern in case 3. The diagnosis was further confirmed by the finding of transbronchial lung biopsy in case 1, and the clinical finding showing ineffectiveness of broad-spectrum antibiotics in case 3. In addition, case 2 came to the hospital with disturbed consciousness, and showed decreased levels of adrenocorticotropic and cortisol hormones. Magnetic resonance imaging revealed enlargement of the anterior pituitary gland and pituitary stalk, which led to the diagnosis of hypophysitis. The two patients with grade 2 or 3 pneumonitis needed to be treated with corticosteroids and a patient with grade 3 hypophysitis received the corticosteroids replacement therapy. The symptoms of case 1, 2 and 3 have been improved by the treatments, but treatments with nivolumab were discontinued. No irAEs-related death was observed in this cohort.

**Table 3:** Immune-related adverse events (irAEs)

	Total	%	Grade 1	Grade 2 ≤
No. of irAE patients	8	72.7		
IrAE event (total)	9		2	7
Infusion reaction	1	9.1	1	0
Itching	1	9.1	1	0
Hypothyroidism	2	18.2	0	2
Pneumonitis	2	18.2	0	2
Hypophysitis	1	9.1	0	1
Rash maculopapular	1	9.1	0	1
Bullous dermatitis	1	9.1	0	1

**Table 4:** Patient details of grade ≥ 2 irAEs and treatment cycles, OR at the time of irAEs onset

Case	irAEs	Treatment cycles	OR	Continuity
1	Grade 3 pneumonitis, grade 2 hypothyroidism	14	SD	Discontinue
2	Grade 3 hypophysitis	18	SD	Discontinue
3	Grade 2 pneumonitis	31	PR	Discontinue
4	Grade 2 hypothyroidism	3	SD	Continue
5	Grade 2 rash maculopapular	17	SD	Continue
6	Grade 2 bullous dermatitis	3	PR	Continue

*OR* objective response, *CR* complete response, *PR* partial response, *SD* stable disease

### Correlation between irAEs and therapeutic effects

**Fig. 3:**
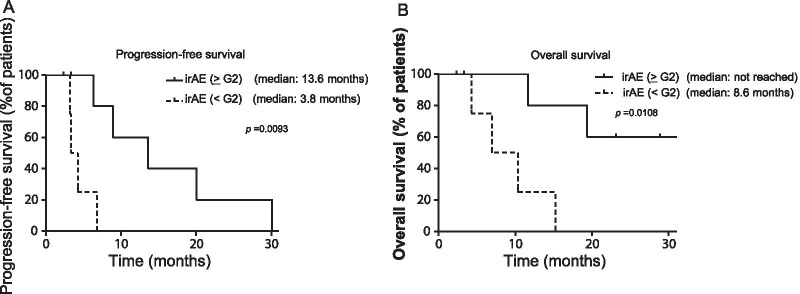
Correlation between irAEs and therapeutic effects. Kaplan–Meier survival curve of PFS (**A**) and OS (**B**). PFS and OS following nivolumab treatment in the grade ≥ 2 irAEs group (n = 6) and grade < 2 irAEs group (n = 5). The median PFS was significantly longer in the grade ≥ 2 irAEs group than in the grade < 2 irAEs group (*p* = 0.0093). OS was significantly longer in the grade ≥ 2 irAEs group than in the grade < 2 irAEs group (*p* = 0.0108)

We next examined the correlation between irAEs and therapeutic effects in patients with MPM treated with nivolumab. We analyzed the therapeutic effects separately for the patients with grade < 2 irAEs or without irAEs (n = 5) and the patients with grade ≥ 2 irAEs (n = 6). Median PFS was significantly longer in the grade ≥ 2 irAEs group (13.6 months) than in the grade < 2 irAEs group (3.8 months; *p* = 0.0093; Fig. 3A). OS was significantly longer in the grade ≥ 2 irAEs group than in the grade < 2 irAEs group (*p* = 0.0108; Fig. 3B).

## Discussion

Nivolumab has been shown to be effective as a second-line treatment of MPM, and the ORR, PFS, and OS were reported to be 24–29%, 2.6–6.1 months, and 17.3 months, respectively [6, 7]. The treatment effects in the current study were similar to previous reports. In terms of the adverse events, there is a report showing that 32.4% of patients treated with nivolumab in the second line had treatment-related adverse events of grade 3 or higher [7]. Our study also showed that treatment-related adverse events of grade 3 or higher were observed in two patients (25.0%), which was less frequent than the previously report, though the number of patients was small.

Programmed death-ligand 1 (PD-L1) expression in lung cancer [15, 16] and microsatellite instability-high (MSI-H) in colorectal cancer [17] are used to predict the therapeutic effects of immune checkpoint inhibitors (ICIs). Among MPM patients, the PD-L1 expression was reported to be negative, positive, and highly positive in 58.2, 41.8, and 9.6% of the patients, and the high PD-L1 expression was associated with worse OS [18]. In another report, two patients were identified as MSI-H among 83 MPM patients [19], however, the correlation between MSI-H and the therapeutic effects of ICIs in MPM is still unclear. Matsuoka et al. recently analyzed a cohort with various types of cancer including MPM, and showed that ORR, OS and PFS were significantly better in the patients with irAEs than in those without irAEs [20]. Although this cohort included only four MPM patients in total 260 cases, none of them developed irAEs, therefore, it was still unclear whether there is a correlation between irAEs and therapeutic effect in patients with MPM. In this study, we examined the eight patients with MPM, and showed the PFS and OS in the patients with irAEs was significantly longer than their counterpart. To our best knowledge, it is a first report identifying the clinical factor which correlates the clinical benefits of ICIs treatment in patients with MPM.

In a phase II study (MERIT study), the treatments of ICIs in 4 out of 34 patients (12%) had to be terminated due to the adverse events [7]. In the present study, the treatments of nivolumab in two patients with pneumonitis and one patient with hypophysitis were discontinued and not rechallenged. On the other hand, there is another report that rechallenge of ICI was found to be effective in 7.4% of patients with various types of cancer, and 28.8% of these patients developed the same irAEs that occurred in the first treatment [21]. These results also support our findings that the ICIs have a potential to show the favorable clinical response in the patients with irAEs, therefore, to prevent the treatment from discontinuing or rechallenge it by careful monitoring and appropriate intervention would improve the outcome in these patients.

Our study has several limitations. Because MPM is a rare disease and nivolumab was approved for patients with MPM in 2018, sample size is small. So we should carefully make a conclusion. It was a retrospective study and conducted in a single institution, so it may contain the selection bias. All histological types were epithelial type, so the correlation between irAEs and therapeutic effects in the other histological types remains unclear.

## Conclusions

This is the first study to report the correlation between irAEs and therapeutic effect of nivolmab in patients with MPM. Because the presence of irAEs may be associated with a favorable PFS and OS, early detection and appropriate management of irAEs would enable us to treat these patients without discontinuation, resulted in improving the therapeutic benefits of this treatment.

## Data Availability

The datasets used and analyzed during the current study are available from the corresponding author on reasonable request.

## Abbreviations

MPM: Malignant pleural mesothelioma
irAEs: Immune-related adverse events
PFS: Progression-free survival
OS: Overall survival
PD-1: Programmed cell death protein 1
ECOG: Eastern cooperative oncology group
PS: Performance status
NCI-CTCAE: National Cancer Institute-Common Toxicity Criteria for Adverse Events
CR: Complete response
PR: Partial response
SD: Stable disease
PD: Progressive disease
mRECIST: Modified response evaluation criteria in solid tumors
RECIST: Response evaluation criteria in solid tumors
ORR: Objective response rate
DCR: Disease control rate
PD-L1: Programmed death-ligand 1
MSI-H: Microsatellite instability-high
ICIs: Immune checkpoint inhibitors
